# Gastric acid response to acute exposure to hypergravity

**DOI:** 10.18632/oncotarget.13969

**Published:** 2016-12-15

**Authors:** Gun Yoon, Hyun-Soo Kim

**Affiliations:** ^1^ Shinsegae Women's Hospital, Daegu, Republic of Korea; ^2^ Department of Pathology, Severance Hospital, Yonsei University College of Medicine, Seoul, Republic of Korea

**Keywords:** hypergravity, gastric acid, gastrin, rat, Pathology Section

## Abstract

The influence of environmental stressors on the pathogenesis of gastrointestinal disease has received increased awareness. Stress affects different physiological functions of the gastrointestinal tract, including gastric acid secretion and mucosal blood flow. Repeated exposures of rapid-onset, highly-sustained hypergravity cause severe physical stress in the pilot. Although the effects of exposure to hypergravity on cardiovascular and cerebral functions have been the subjects of numerous studies, crucial information regarding pathophysiological changes in the gastrointestinal tract following hypergravity exposure is lacking. In this study, we investigated the effects of acute exposure to hypergravity on gastric secretory activity and gastrin release. Male Sprague-Dawley rats were exposed to +10Gz three times for 3 min. Gastric juice and blood were collected. The volume and total acidity of gastric juice, and the plasma gastrin level was measured. Acute exposure to +10Gz significantly decreased the gastric juice parameters. The gastric juice volume and total acidity of hypergravity-exposed rats were 3.54 ± 0.32 mL/100 g and 84.90 ± 5.17 mEq/L, respectively, which were significantly lower than those of the nonexposed rats (4.62 ± 0.39 mL/100 g and 97.37 ± 5.42 mEq/L; *P* < 0.001 and *P* < 0.001, respectively). In contrast, plasma gastrin level was not significantly altered following hypergravity exposure. We demonstrated that acute exposure to hypergravity led to a significant decrease in the gastric juice volume and acidity but did not alter the plasma gastrin level.

## INTRODUCTION

In jet fighter aircrafts, pilots experience exceptionally high inertial forces in the head-to-foot direction (+Gz) because of the gravitational acceleration these airplaines can achieve. With the development of modern, lightweight, high-thrust aircraft, acceleration limits are mainly determined by the physiology of the pilot, rather than the performance or structural limitations of the airplane. Such environments of rapid-onset, highly-sustained +Gz cause severe physical stress in the pilot [1]. Therefore, it is important to determine any alterations related to hypergravity exposure. Numerous studies on human exposure to hypergravity have demonstrated various pathophysiological effects, including changes in cerebral, coronary, and renal blood flow, endocrine reactions, and cardiovascular reflexes [2–6].

Pathophysiological effects of hypergravity mainly include structural and functional alterations in the visceral organs, especially, impaired visceral blood flow [3, 7, 8]. A reduction in the visceral blood flow may result from the combination of hypergravity-induced cardiovascular reflex responses with emotional stress causing sympathetic vasoconstriction and increasing the peripheral resistance of visceral vascular beds. Exposure to hypergravity leads to significantly decreased blood flow to the spleen, pancreas, liver, and kidneys, likely maintain sufficient blood flow to the brain and heart [7, 9, 10].

The gastrointestinal system is especially vulnerable to acute or chronic stress, as demonstrated by the stress-induced changes in gastric acid secretion, motility, mucosal permeability, barrier function, visceral sensitivity, and mucosal blood flow [11]. Although the effects of exposure to hypergravity on cardiovascular and cerebral functions have been extensively studied, the potential pathophysiological effects on the gastrointestinal tract are unknown. Furthermore, alteration in gastric acid secretion after exposure to hypergravity has not yet been documented. In this study, we investigated the effects of acute exposure to hypergravity on gastric secretory activity and gastrin release in rats. We used pylorus ligation technique to induce gastric acid hypersecretion. We measured gastric juice parameters, including volume, acidity, and pH, and plasma gastric level in hypergravity-induced rats. Our observations suggest that hypergravity exposure significantly decreased gastric acid secretion and acidity.

## RESULTS

### Effects of pylorus ligation and hypergravity exposure on gastric juice parameters

The rats were subjected to pylorus ligation as reported by Shay et al. [12, 13]. We first analyzed the impacts of pylorus ligation on gastric juice parameters. The gastric juice volume of pylorus-ligated rats (4.32 ± 0.67 mL/100 g) was significantly higher than that of nonligated rats (3.83 ± 0.54 mL/100 g; *P* = 0.014). Likewise, the total acidity of pylorus-ligated rats (94.57 ± 8.17 mEq/L) was significantly higher than that of nonligated rats (87.71 ± 6.82 mEq/L; *P* = 0.006). On the other hand, the gastric juice pH of pylorus-ligated rats was 3.00±0.29, which was lower than that of nonligated rats (3.17 ± 0.32), albeit not significantly (*P* = 0.083). These findings showed that pylorus ligation elevated the volume and acidity of gastric juice.

**Figure 1:**
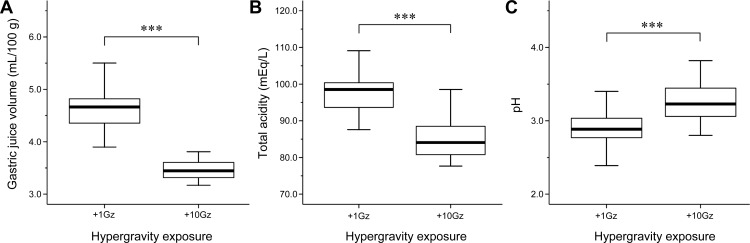
Effects of hypergravity exposure on gastric juice parameters Box-and-whisker diagram. The band inside each box indicates the median value of each group. Acute exposure to hypergravity (+10Gz) significantly decreases **A.** gastric juice volume and **B.** total acidity and increases **C.** gastric juice pH. One-way ANOVA was used to compare means of samples among groups. ***P < 0.001.

We next examined the influence of hypergravity exposure on gastric juice parameters. The gastric juice volume of hypergravity-exposed rats (3.54 ± 0.32 mL/100 g) was significantly lower than that of nonexposed rats (4.62 ± 0.39 mL/100 g; *P* < 0.001; Figure 1A). Similarly, the total gastric juice acidity of hypergravity-exposed rats (84.90 ± 5.17 mEq/L) was significantly lower than that of nonexposed rats (97.37 ± 5.42 mEq/L; *P* < 0.001; Figure 1B). In contrast, the gastric juice pH of hypergravity-exposed rats (3.28±0.26) was significantly higher than that of nonexposed rats (2.89 ± 0.23; *P* < 0.001; Figure 1C). These results indicated that hypergravity exposure significantly reduced the volume and acidity of gastric juice.

**Figure 2:**
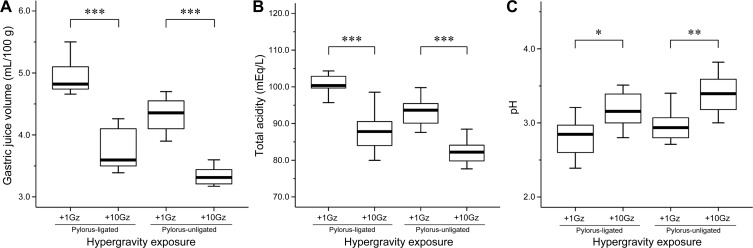
Effects of pylorus ligation and hypergravity exposure on gastric juice parameters Box-and-whisker diagram. The band inside each box indicates the median value of each group. Acute exposure to hypergravity (+10Gz) significantly decreases **A.** gastric juice volume and **B.** total acidity and increases **C.** gastric juice pH in both pylorus-ligated and pylorus-nonligated rats. One-way ANOVA was used to compare means of samples among groups. *P < 0.05; **P < 0.01; ***P < 0.001.

We then compared the gastric juice parameters among four groups of rats classified based on hypergravity exposure and pylorus ligation. The values of gastric juice volume, total acidity, and pH of each group are shown in Table 1. In pylorus-ligated rats, hypergravity exposure significantly decreased gastric juice volume (*P* < 0.001; Figure 2A) and total acidity (*P* < 0.001; Figure 2B), while it increased gastric juice pH (*P* = 0.012; Figure 2C). Similarly, in nonligated rats, the values of gastric juice volume and total acidity of the +10Gz-exposed rats were significantly lower than those of the nonexposed rats (*P* < 0.001 and *P* < 0.001; Figure 2A and Figure 2B, respectively). The gastric juice pH of +10Gz-exposed rats was significantly higher than that of the nonexposed rats (*P* = 0.002; Figure 2C). These results indicated that hypergravity exposure significantly reduced the volume and acidity of gastric juice regardless of pylorus ligation.

**Table 1:** Effect of hypergravity exposure on gastric juice parameters

Group	Gastric juice volume (mL/100 g)	Total acidity (mEq/L)	pH
Pylorus-ligated/+1Gz	4.91±0.26	101.32±3.56	2.82±0.25
Pylorus-ligated/+10Gz	3.74±0.33	87.81±5.19	3.17±0.22
Nonligated/+1Gz	4.32±0.26	93.42±3.83	2.96±0.21
Nonligated/+10Gz	3.34±0.14	81.99±3.28	3.38±0.26

### Effects of pylorus ligation and hypergravity exposure on plasma gastrin concentration

We observed that neither pylorus ligation nor hypergravity exposure affected gastric juice parameters. There was no significant difference between the plasma gastrin concentration of pylorus-ligated rats (98.82 ± 4.77 pg/mL) and that of nonligated rats (98.47 ± 3.42) (*P* = 0.792). Although the plasma gastrin concentration in hypergravity-exposed rats (97.83 ± 4.45 pg/mL) was lower than that in nonexposed rats (99.45 ± 3.66 pg/mL), the difference was not statistically significant (*P* = 0.218).

We also analyzed differences in plasma gastrin concentration in the four groups subjected to hypergravity exposure and/or pylorus ligation. The plasma gastric concentration of each group are shown in Table 2. The plasma gastrin concentration of the pylorus-ligated, +10Gz-exposed rats was 97.72 ± 5.44 pg/mL, which was not significantly different from that of pylorus-ligated, nonexposed rats (99.91 ± 3.96 pg/mL; *P* = 0.646). Similarly, in the nonligated group, the difference in plasma gastrin concentration between +10Gz-exposed (97.95 ± 3.48 pg/mL) and nonexposed (98.99 ± 3.47 pg/mL) rats was not significantly different (*P* = 0.944).

**Table 2:** Effect of hypergravity exposure on plasma gastrin concentration

Group	Plasma gastrin concentration (pg/mL)
Pylorus-ligated/+1Gz	99.91±3.96
Pylorus-ligated/+10Gz	97.72±5.44
Nonligated/+1Gz	98.99±3.47
Nonligated/+10Gz	97.95±3.48

## DISCUSSION

We have investigated the molecular, biochemical, and morphological alterations of heart, liver, and kidney due to acute exposure to hypergravity [14–16]. We used blood or tissue samples obtained from mice or rats after hypergravity exposure to observe significant alterations in the expression of molecular and/or biochemical parameters that reflect hypoxia and/or reperfusion injury. Considering that acute exposure to hypergravity not only affects the brain and heart, but also drastically reduces visceral blood flow, it can be assumed that cellular or tissue injury may occur due to the hypoxia caused by reduced blood flow in most splanchnic organs [10]. If such an injury occurs repeatedly, clinically significant diseases can arise in jet fighter pilots, who are inevitably exposed to repeated episodes of hypergravity. In this regard, the purpose of studying the hypergravity-induced cellular or tissue injury of visceral organs is to predict and prevent diseases that might be caused by hypergravity exposure, thereby helping jet fighter pilots to maintain their health while serving their duties. Acute exposure to hypergravity reportedly causes substantial physical and mental stress, which is one of the major factors causing various gastrointestinal diseases [11]. Since gastrointestinal disorders, such as dyspepsia, abdominal discomfort, abdominal pain, and nausea, which may occur during flight, cause significant inconveniences to jet fighter pilots and can determine the success of their duties, the significance of these diseases cannot be overlooked [11]; however, only few studies on this matter have been conducted. We hypothesized that acute exposure to hypergravity would exert significant impacts on the physiological function of the stomach, and that such impacts would lead to changes in gastric secretory activities. To prove this hypothesis, we analyzed plasma gastrin concentration as well as gastric juice parameters that reflect acid secretion status in hypergravity-exposed rats.

In this study, we observed that gastric juice parameters, including gastric juice volume and acidity, of hypergravity-exposed rats were significantly lower than those of nonexposed rats. This indicates that acute exposure to hypergravity inhibits gastric acid secretion. To the best of our knowledge, no study on changes in gastric acid secretion resulting from hypergravity exposure has been conducted. We measured the plasma concentration of gastrin in hypergravity-exposed rats to examine whether the significant decreases in the gastric juice parameters were caused by a decline in gastrin release due to hypergravity. No significant difference was found in the plasma gastrin concentration between hypergravity-exposed and nonexposed rats. Thus, it is unlikely that the decreases in gastric juice volume and acidity due to hypergravity exposure were caused by gastrin. We assumed that the reduction in gastric acid secretion due to hypergravity was caused by a decline in and blood flow to the stomach. The fact that hypergravity exposure causes a reduction in visceral blood flow has been proven in previous studies. [3, 7–10]. Activation of the sympathetic nervous system due to hypergravity exposure and splanchnic vasoconstriction due to increased catecholamine secretion from the adrenal gland lead to decreased blood flow to the visceral organs, including the stomach. Moreover, we cannot exclude that secretion may have been reduced as visceral organs were pushed downwards due to hypergravity, imposing a pressure on the tissues that secrete gastric acid. Further investigations should be conducted to elucidate the mechanism of hypergravity-induced gastric acid hyposecretion.

Pylorus ligation is an experimental procedure stimulating gastric acid secretion, and is used to examine the impacts of a certain condition or drug on gastric acid secretion. We observed that gastric juice volume and acidity were significantly higher in pylorus-ligated than in nonexposed rats. This result is consistent with those of previous studies on gastric acid hypersecretion following pyloric ligation [13, 17]. On the other hand, pylorus ligation did not have significant impacts on plasma gastrin concentration. This finding too is consistent with that of a previous study, that indicated that pyloric ligation stimulates gastric acid secretion, but has no significant impact on the plasma gastrin level [17]. We observed that hypergravity exposure significantly decreased gastric juice parameters regardless of pyloric ligation.

In conclusion, we observed that hypergravity exposure significantly reduced the volume and acidity of gastric juice in rats. In addition, we found that pylorus ligation significantly increased gastric acid secretion, consistent with the finding of previous studies. No significant alteration in plasma gastrin concentration following hypergravity exposure indicates that the reduction in gastric juice parameters caused by hypergravity exposure cannot be attributed to changes in gastrin release. Thus, further investigation on the causes of hypergravity-induced gastric acid hyposecretion is required.

## MATERIALS AND METHODS

### Experimental animals

Sprague-Dawley rats, eight weeks of age and weighing 200–220 g, were purchased. The rats fed standard laboratory rat chow, provided with free access to water, and maintained on a 12-h light/dark cycle under temperature and moisture levels controlled at 20–25°C and 40–45%, respectively. To avoid any effect of unfavorable factors including fear and stress, the rats were acclimatized to the rearing environment for 7 d.

### Pylorus ligation and hypergravity exposure

The rats were fasted for 24 h, with free access to water. Under light anesthesia, laparotomy was performed through a midline incision of approximately 3 cm. The pyloric portion of the stomach was gently mobilized and occluded with a 4-0 silk ligature around the pyloric sphincter [12]. The incision was closed with 3-0 silk sutures. The pylorus-ligated rats were randomly assigned to two groups. The rats of the first group (n=10) were exposed to +10Gz three times for 3 min (onset rate, +1Gz/s; interval between exposure, 5 min) using a small-animal centrifuge. Each rat was placed into a cylindrical plastic restraint device that, when mounted in a centrifuge, allowed +Gz to be delivered along the rostro-caudal axis. After the rats were secured, the restraint device was clamped to the end of the centrifuge arm, which allows one degree of freedom, thereby ensuring that the net G field was perpendicular to the floor of the restraint device. The rats of the second group (n=10) were placed in the centrifuge arm and underwent a process similar to the one described above, but they were not exposed to hypergravity. The behavior of the rats was monitored with a charge-coupled device camera throughout the centrifugation experiments. The Institutional Animal Care and Use Committee of the Republic of Korea Air Force Aerospace Medical Center (Cheongju, Chungcheongbuk-do, Republic of Korea) approved all experimental procedures involving the animals.

### Measurement of the gastric juice parameters

Three hours after pylorus ligation, the rats were euthanized. The abdomen was opened, the stomach was removed and the gastric content was collected and centrifuged at 8,000×*g* for 10 min at 25°C. The volume (mL/100 g), total acidity (mEq/L), and pH of the gastric juice were determined. The total acidity was determined by titration to pH 7.0 with 0.01 N sodium hydroxide using phenolphthalein as an indicator. The pH was measured using a digital pH meter.

### Measurement of plasma gastrin concentration

Just before stomach removal, blood was collected from the abdominal aorta. The heparinized blood was centrifuged at 3,000×*g* for 10 min and the plasma was kept at −20°C until analysis. The plasma concentration of gastrin was determined using a commercially available enzyme-linked immunosorbent assay kit (Abcam, Cambridge, UK).

### Statistical analysis

All values are provided as the mean ± standard error. Differences between the groups were assessed using Student's *t*-test or one-way analysis of variance (ANOVA) followed by Tukey's multiple range tests. Statistical analyses were conducted using PASW Statistics for Windows (version 18.0; Armonk, NY, USA). Statistical significance was defined as a *P* value less than 0.05.
